# Supplementary material for: Light-dependent grazing can drive formation and deepening of deep chlorophyll maxima
Source: Nat Commun. 2019 Apr 29;10:1978. doi: 10.1038/s41467-019-09591-2 (PMC6488668; doi:10.1038/s41467-019-09591-2)
Supplement: Supplementary file 1 — Supplementary Information [file 41467_2019_9591_MOESM1_ESM.docx]

**ONLINE-ONLY SUPPLEMENTARY MATERIAL: Light-Dependent Grazing Can Drive Formation and Deepening of Deep Chlorophyll Maxima**

Authors:

Holly V. Moeller et al.

**
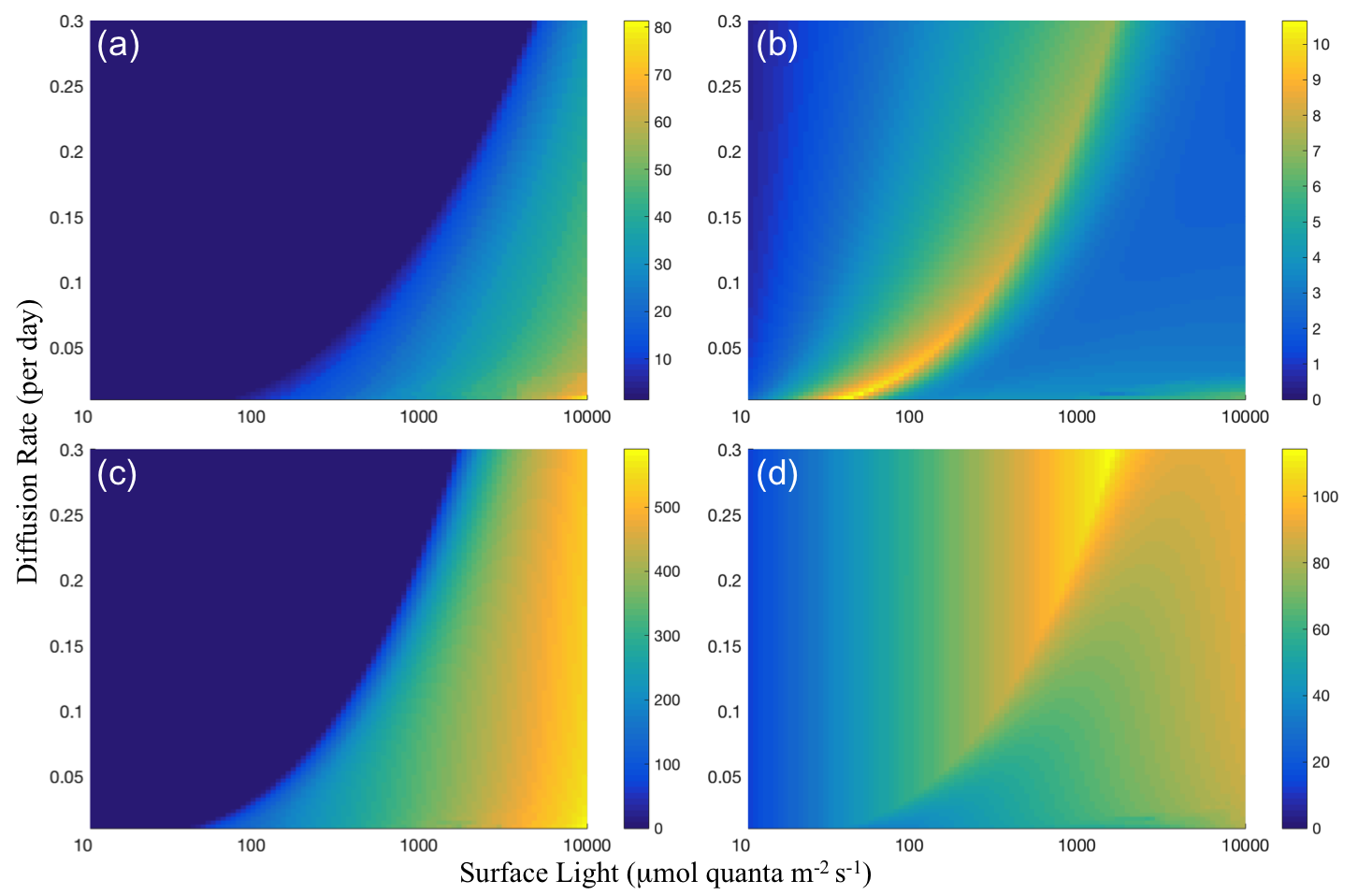
**

**Supplementary Figure 1. One-dimensional model output as a function of surface irradiance and diffusion.** Heatmaps show DCM depth **(a)**, phytoplankton biomass at the DCM **(b)**, total microzooplankton biomass integrated over the entire water column **(c)**, and total phytoplankton biomass **(d)**. Parameter values are *k*_0_ = 0.001, *k*_P_ = 0.1, *k*_Z_ = 0.0005, *p* = 1, *l* = 0.5, *g* = 0.05, *e* = 1, *m* = 0.05, *H*_P_ = 0.5, and *H*_Z_ = 50.


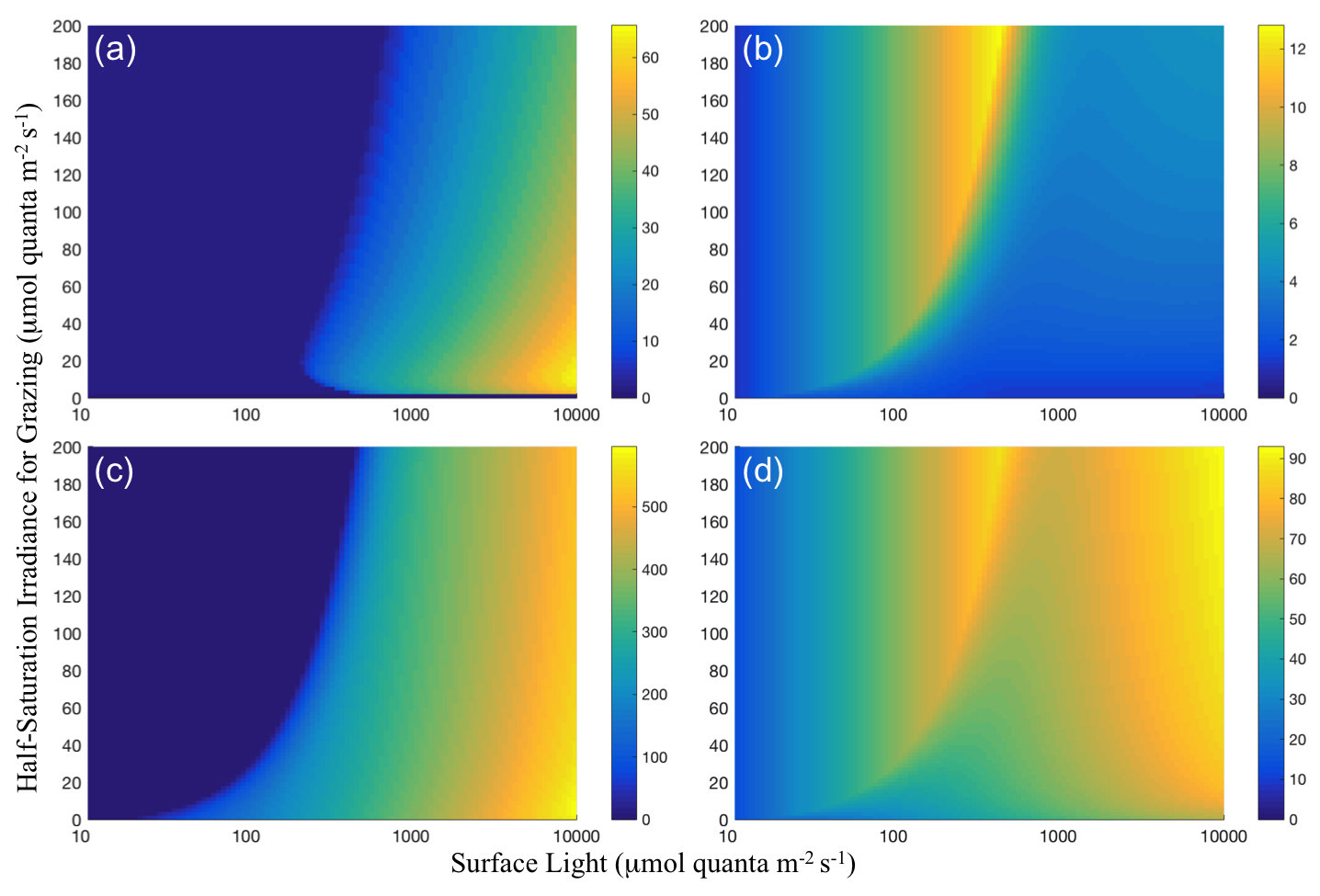


**Supplementary Figure 2. One-dimensional model output as a function of surface irradiance and grazing half-saturation.** Heatmaps show DCM depth **(a)**, phytoplankton biomass at the DCM **(b)**, total microzooplankton biomass integrated over the entire water column **(c)**, and total phytoplankton biomass **(d)**. Parameter values are *k_0_* = 0.001, *k*_P_ = 0.1, *k*_Z_ = 0.0005, *p* = 1, *l* = 0.5, *g* = 0.05, *e* = 1, *m* = 0.05, *H*_P_ = 0.5, and *D* = 0.05.

**Supplementary Figure 3. Comparison of alternative 1-D model formulations.** Plots show total phytoplankton and heterotroph biomass (left column), and DCM depth and phytoplankton biomass at the DCM (right column) for (1) a model with a Holling Type I predator functional response (top row, panels **a** and **b**), (2) a model with a linear relationship between light and grazing (middle row, panels **c** and **d**), and (3) a model in which light affects handling time (bottom row, panels **e** and **f**). Note that all results are qualitatively identical to those obtained with a Holling Type II predator functional response and a saturating dependence of attack rate on light availability (main text). Specifically, when light levels are sufficiently high, a DCM forms. At this DCM, the abundance of phytoplankton is approximately constant even as light continues to increase, though the total biomass of both phytoplankton and heterotrophs increases.

**Supplementary Figure 4. Annual mean percent change in DCM depth between COBALT model runs.** Percent change is calculated as (modified DCM depth – control DCM depth) / control DCM depth * 100, so positive numbers (red end of the scale) represent a deepening of the DCM with light-dependent grazing, and negative numbers (blue end of the scale) represent a shoaling of the DCM with light-dependent grazing.

**Supplementary Figure 5. Comparison between COBALT predicted DCM depths and Mignot *et al.* (2014) satellite-derived observations.** Histograms of **(a)** percent differences (observation – model output) / observation * 100) and **(b)** root mean square differences (sqrt((observation-model output)^2^ / observation^2^) are shown for the unmodified (control) COBALT model (blue) and the modified (with light-dependent grazing) COBALT model (red). The ideal expectation is indicated with a black vertical line at x = 0. Two-sample *z*-tests were used to demonstrate statistically significantly better fits for the COBALT model modified to include light-dependent grazing (percent difference: μ_ctrl_ = 7.775, μ_LDG_ = -3.096, *z*-statistic = 34.227, *p* < 0.001; RMS: μ_ctrl_ = 34.308, μ_LDG_ = 24.943, *z-*statistic = 24.806, *p* < 0.001).

**Supplementary Figure 6. COBALT modification has little impact on other model outputs.** We compared output from the unmodified (control, blue) and modified (to include light-dependent grazing, red) COBALT model to World Ocean Atlas surface nitrate (top row, panels **a** and **b**) and SeaWiFS surface chlorophyll-*a* (bottom row, panels **c** and **d**) data. Percent differences (left column), calculated as (Ref. Data – Model Output) / Ref. Data, were not significantly different between model runs (*z*-test, p = 0.6127 for nitrate, p = 0.7958 for chl-*a*). Boxplots (panels **a** and **c**) show median (heavy black line), upper and lower quartiles (upper and lower box edges), and minimum and maximum values excluding outliers (extent of whiskers) for unmodified (control; blue) and modified (with light-dependent grazing; red) COBALT model runs. Although root mean square differences in nitrate **(c)**, calculated as sqrt((Reference Data – Model Output)^2^), were significantly higher for the modified model (*z*-test, p < 0.05), the median RMSD was lower for the modified model, suggesting that the inclusion of light-dependent grazing tended to improve the global fit, but cause greater deviations from Atlas data at extremes. Both mean and median RMSD in chl-*a* **(d)** were greater for the modified COBALT model (*z*-test, p < 0.05), but these deviations were small in comparison to the overall range of chl*-a* concentrations in the surface ocean.**
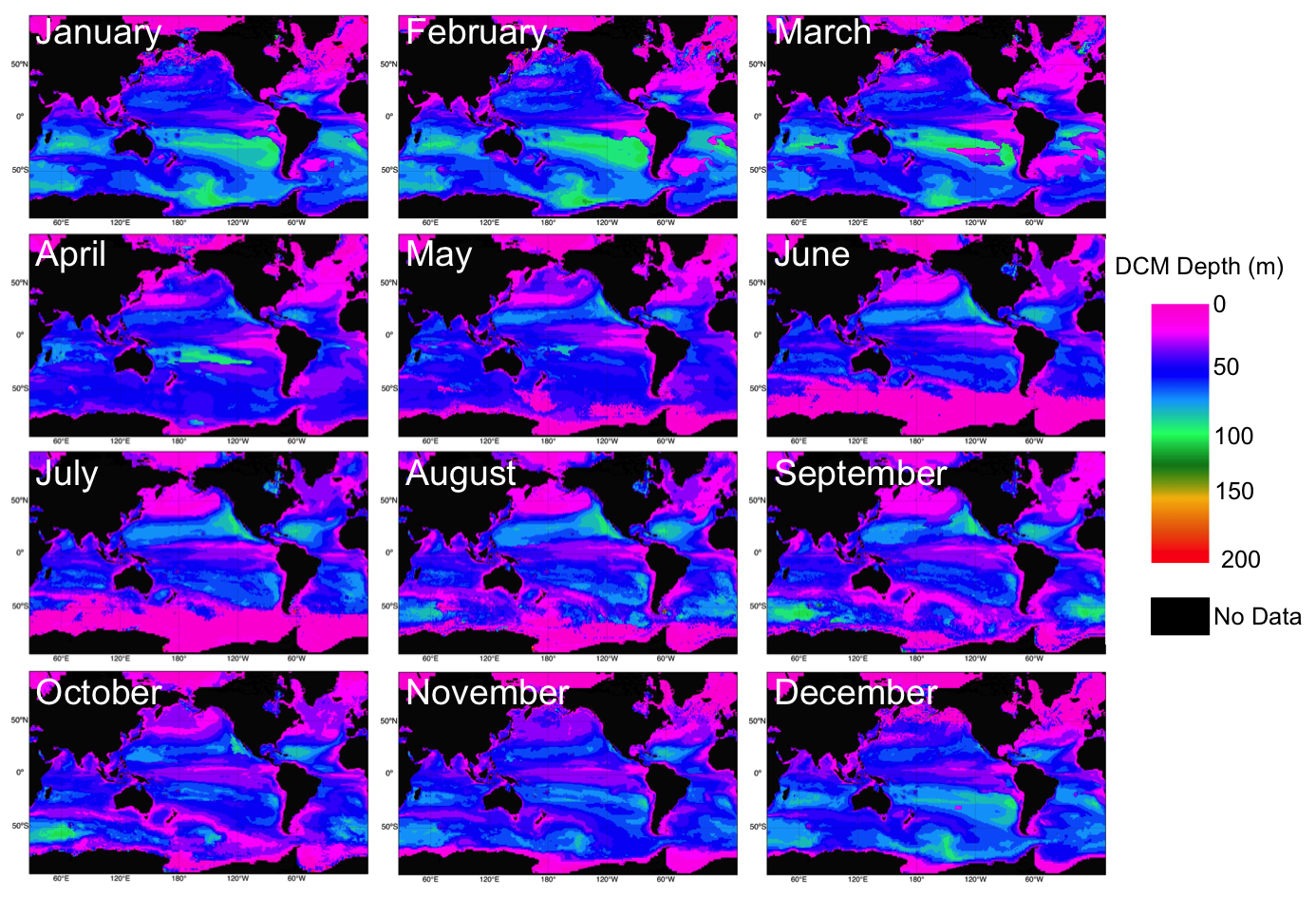
**

**Supplementary Figure 7. Unmodified COBALT model climatology of DCM depth.** Each panel represents the average value for a one-month interval.

**
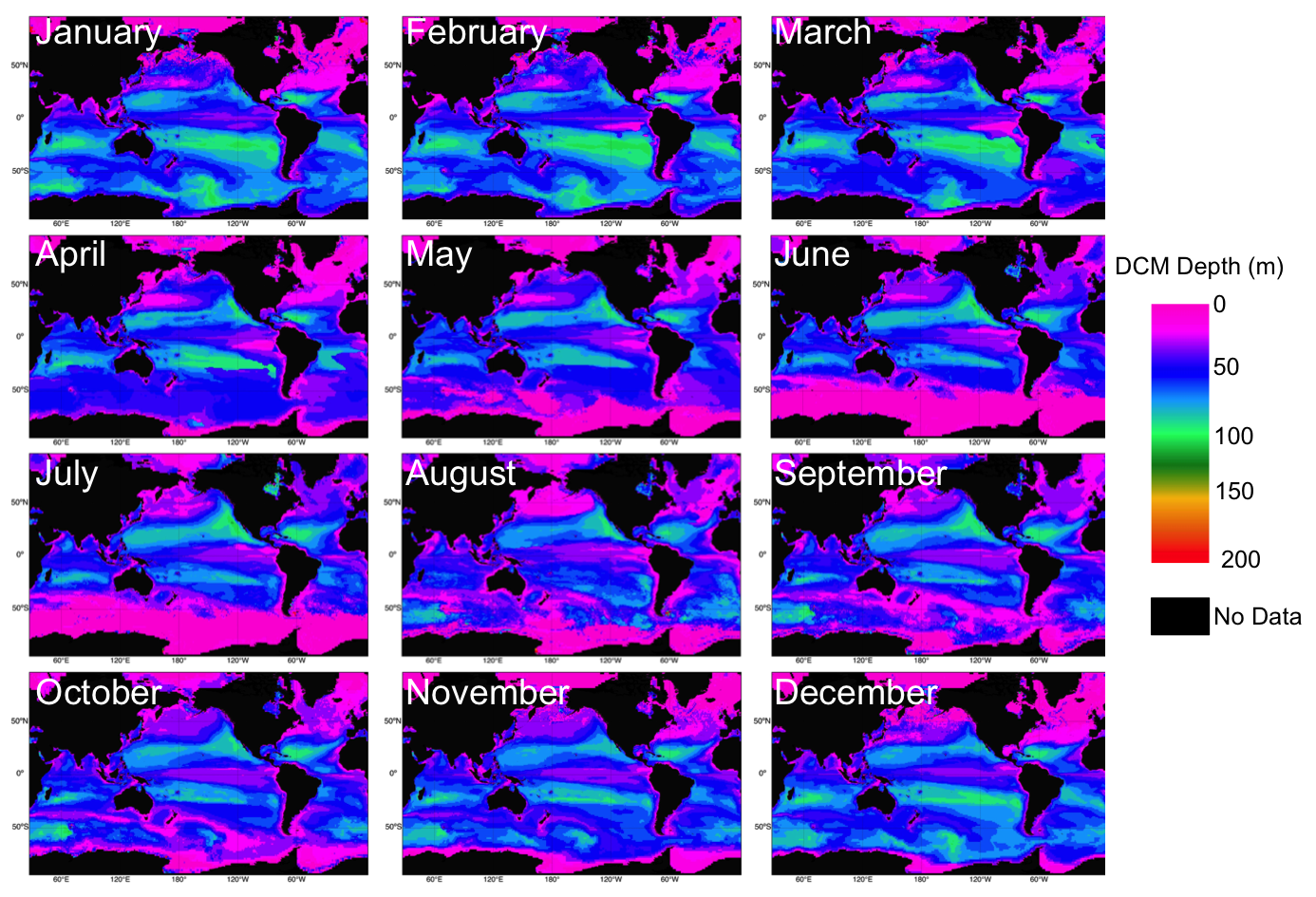
**

**Supplementary Figure 8. Climatology of DCM depth projected by the COBALT model with light-dependent grazing.** Each panel represents the average value for a one-month interval.

**
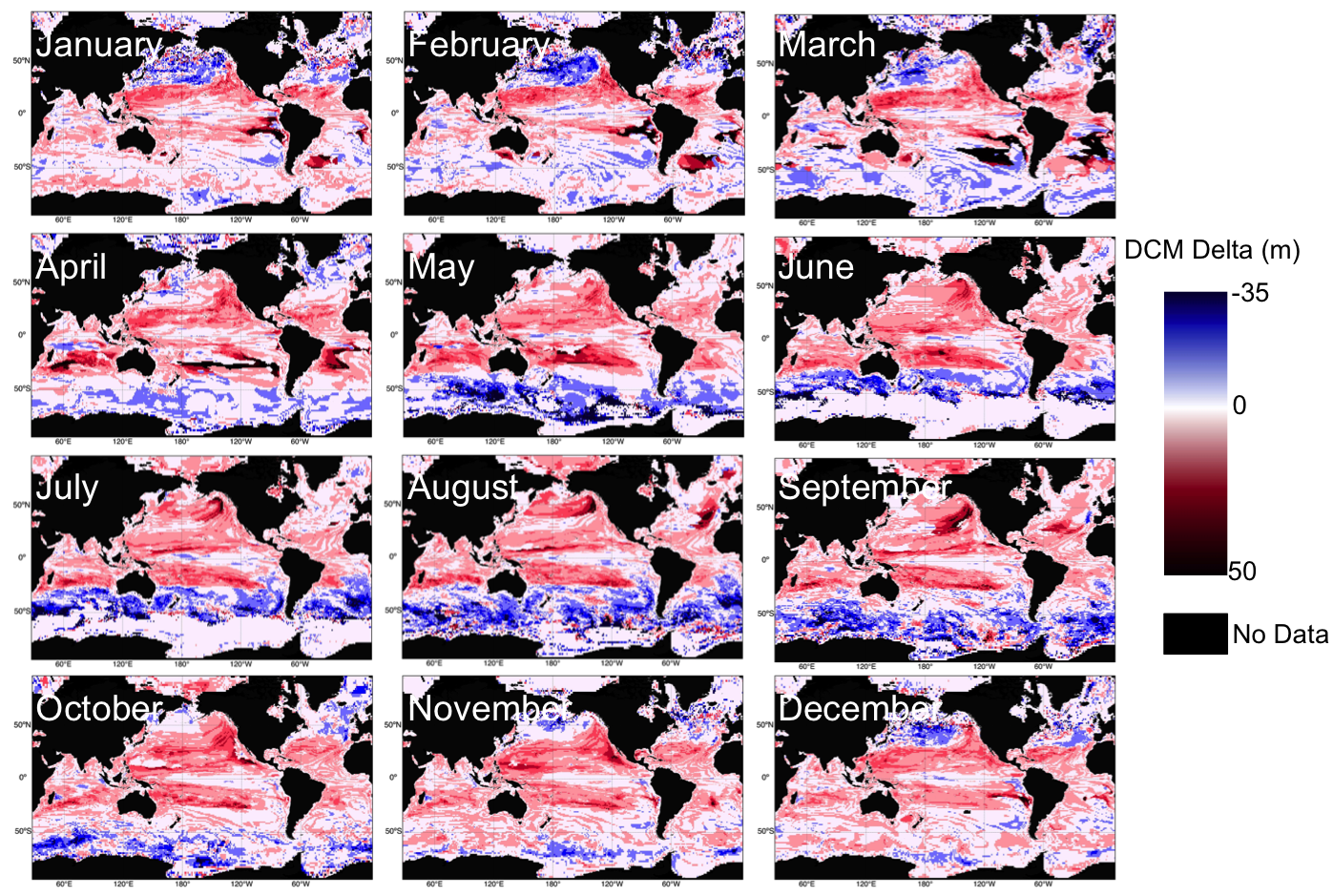
**

**Supplementary Figure 9. Differences in DCM depth between COBALT model runs.** Differences are calculated as the depth of the DCM in the modified model minus the depth of the DCM in the control run. Each panel represents the average value for a one-month interval. Warmer colors indicate that the DCM was deeper in the modified model; cooler colors indicate a shoaling of the DCM with light-dependent grazing.

**Supplementary Figure 10. Comparison of nutricline and DCM depths across COBALT model runs.** Boxplots show median (heavy black line), upper and lower quartiles (upper and lower box edges), minimum and maximum values excluding outliers (extent of whiskers), and outliers (circles) for unmodified (control; blue) and modified (with light-dependent grazing; red) COBALT model runs. Stars indicate means. Inclusion of light-dependent grazing did not significantly deepen the nutricline **(a)**, nor did it cause DCMs to deepen below the nutricline **(b)**. These results suggest that observation of nutriclines that are deeper than DCMs does not categorically eliminate the possibility that light-dependent grazing is contributing to DCM location.
